# Residue of Organophosphate Esters (OPEs) in the Crustacean from Southeast China and Its Dietary Exposure Risk Assessment

**DOI:** 10.3390/jox16020058

**Published:** 2026-03-27

**Authors:** Hai-Tao Shen, Jian-Long Han, Xiao-Min Xu, Xiao-Dong Pan

**Affiliations:** 1 Zhejiang Provincial Center for Disease Control and Prevention, Room No. 3-201, Bin-Sheng Road No. 3399, Binjiang District, Hangzhou 310051, China; 2 NHC Specialty Laboratory of Food Safety Risk Assessment and Standard Development, Hangzhou 310051, China

**Keywords:** OPEs, crustacean, organic contaminant, dietary exposure, risk assessment

## Abstract

This study presents a comprehensive investigation of OPE residues, distribution patterns, and dietary exposure risks in crustaceans from southeast China. OPEs were detected in over 90% of samples, with mean total concentrations (ΣOPEs) of 5.80 μg/kg wet weight (ww) in freshwater shrimp, 6.52 μg/kg ww in marine prawn, and 1.25 μg/kg ww in marine crab. Tributyl phosphate (TiBP), triethyl phosphate (TEP), and tris(2-chloroethyl) phosphate (TCEP) emerged as the dominant congeners, accounting for 68.1% of ΣOPEs, which indicates inputs from industrial emissions, plastic waste leaching, and aquaculture equipment. Spatial analysis revealed striking regional differences: coastal industrial cities (Zhoushan, Taizhou) exhibited ΣOPE levels up to 12-fold higher than inland mountainous areas (Quzhou, Lishui), while no significant temporal variations were observed. Human health risk evaluation, based on estimated daily intake (EDI) and target hazard quotient (THQ), demonstrated negligible non-carcinogenic risks for the general population (HI < 1), though children and frequent seafood consumers have slightly elevated exposure. These findings indicate the value of crustaceans as bioindicators for OPE contamination and require long-term monitoring of emerging OPEs and their synergistic effects with co-occurring pollutants.

## 1. Introduction

Organophosphate esters (OPEs) have emerged as a significant class of environmental contaminants, widely adopted as substitutes for brominated flame retardants and broadly utilized as plasticizers in diverse industrial and consumer products [1]. This extensive application facilitates their continuous release into the environment, leading to their presence in matrices such as water, sediment, air, and biota. The ubiquitous distribution of OPEs is a growing concern due to their ecotoxicological and human health impacts, including neurotoxicity, reproductive and developmental toxicity, carcinogenicity, and endocrine disruption [2].

In China, OPE contamination is particularly evident in aquatic environments. Studies across major water bodies like the Yangtze River, Pearl River, Yellow Sea, East China Sea, and South China Sea consistently report the presence of OPEs [2,3]. For instance, total OPE concentrations in the surface water of the Pearl River can range from 117.5 to 854.8 ng/L, with chlorinated OPEs constituting a dominant fraction (79 ± 15%) [4]. Within the Yangtze River basin, OPE concentrations in surface water range from 22.86 to 1398 ng/L, typically increasing from upstream to downstream areas and indicating significant influence from urban and industrial activities [5,6]. Coastal regions, such as the Beibu Gulf in the South China Sea, also exhibit substantial OPE contamination, with summer concentrations (34.2~1227 ng/L) being significantly higher than winter levels (20.6~840 ng/L), suggesting dynamic release and transport mechanisms influenced by environmental factors [7]. Common OPE compounds such as Triethyl phosphate (TEP), Tri(2-chloroethyl) phosphate (TCEP), and Tri (chloropropyl) phosphate (TCPP) are frequently detected across these regions [8]. The main sources of these pollutants include discharges from wastewater treatment plants, industrial effluents, and specific activities like fishing.

Crustaceans, as main components of aquatic food webs and widely consumed seafood, serve as crucial indicators for environmental monitoring and human exposure assessments. Their benthic or sessile lifestyles often render them reliable bioindicators of environmental contamination [9]. Studies have demonstrated significant OPE bioaccumulation in various crustacean species. For instance, crayfish from the Jianghan Plain, China showed a 100% detection rate for OPEs, with TEP identified as the predominant contaminant [10]. Notably, farmed crayfish exhibited higher OPE concentrations compared to wild counterparts, suggesting potential influences of aquaculture practices on pollutant uptake [10]. In marine environments, certain crustaceans, such as barnacles, tend to accumulate higher proportions of chlorinated OPEs (Cl-OPEs), which may be attributed to species-specific metabolic efficiencies [11]. The overall bioaccumulation of OPEs in aquatic organisms is a complex process mediated by pollutant concentration, hydrophobic properties, and metabolic capacity [12]. Specific OPEs, including TEP, TCrP, TCIPP, and TCEP, are of particular concern due to their bioaccumulation potential in crustaceans.

Human exposure to OPEs primarily occurs through dietary intake, dust ingestion, inhalation, and dermal contact. Consumption of contaminated seafood constitutes a significant exposure pathway [13]. Although probabilistic risk assessments, such as Monte Carlo simulations, often suggest a low health risk from OPEs through dietary consumption for the general population, particular attention is still warranted for sensitive and specialized populations. Children, teens, and adults in coastal areas, along with individuals who consume large quantities of seafood, may face elevated exposure risks. The increasing global consumption of aquatic products, particularly in coastal regions, further amplifies the potential for rising OPE exposure levels in human populations.

Although many studies have been reported, we still lack critical information about organophosphate esters (OPEs) in crustaceans from the southeast coast of China. Therefore, this study aims to investigate the residue levels of OPEs in crustaceans from southeast China, characterize their compositional profiles, assess their bioaccumulation potential, and evaluate human health risks. This research will provide scientific data to inform effective environmental management strategies and public health protection measures.

## 2. Materials and Methods

### 2.1. Sample Collection

**Figure 1:**
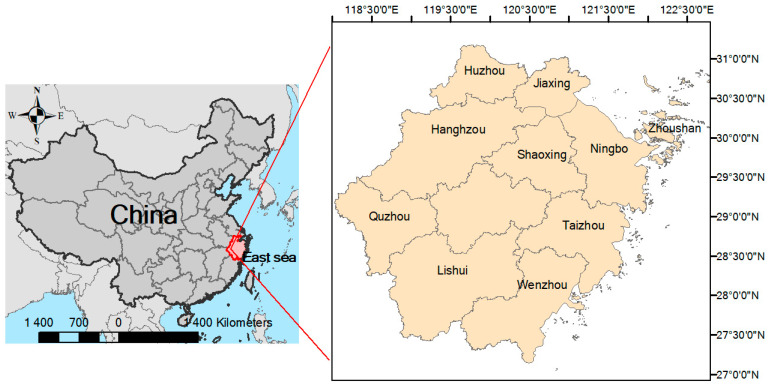
The simple map of sampling areas.

Representative crustacean species, such as shrimp, crabs, and shellfish were sampled from local markets to thoroughly investigate OPEs in crustaceans from southeast China. These species are not only ecologically significant but also form a substantial part of the local diet. Sampling areas were selected considering possible OPE contamination, including estuaries heavily influenced by human activities, and aquaculture areas (Figure 1). Samples, including freshwater shrimps (*n* = 239), marine crabs (*n* = 84) and marine prawns (*n* = 210), were collected from 2024 to 2025. Freshwater shrimps were *Macrobrachium rosenbergii*, *Macrobrachium nipponense*, and *Exopalaemon carinicauda*. Marine prawns were *Penaeus vannamei*, *Penaeus japonicus*, and *Litopenaeus vannamei.* Marine crabs were *Portunus trituberculatus*, *Portunus sanguinolentus*, and *Scylla serrata*. All edible parts of samples were cut, grounded, and stored at −20 °C until the laboratory analysis.

### 2.2. Instrumental Analysis (Sample Extraction, UHPLC-MS/MS Analysis)

The collected crustacean samples were treated with a preparation process prior to instrumental analysis. Samples will be thoroughly rinsed with ultrapure water, and edible portions (e.g., muscle, hepatopancreas) will be carefully dissected, homogenized, and stored at −20 °C until analysis. To prevent background contamination, all glassware and laboratory equipment will be meticulously cleaned with organic solvents (acetone and methanol) and baked at high temperatures (450 °C) for 4 h.

Sample extraction was conducted via acetonitrile extraction followed by solid-phase extraction (SPE) purification, as detailed below. Briefly, 5.0 g of homogenized sample was spiked with 10 μL of 1 μg/mL internal standard solution, and then mixed with 10 mL of acetonitrile containing 0.5% (*v*/*v*) formic acid. The resulting mixture was vortexed for 2 min and subsequently sonicated for 15 min to facilitate analyte extraction. After adding 2.5 g of sodium chloride (NaCl), the mixture was vortexed again for 2 min and centrifuged at 8000× *g* for 10 min. The upper supernatant was carefully collected for further purification. Purification of the collected supernatants was performed using Oasis PRiME HLB SPE columns (200 mg/6 cc) (Waters Corporation, Milford, MA, USA). Prior to sample loading, each SPE column was preconditioned with 2 mL of acetonitrile. A 5 mL aliquot of the supernatant was then loaded onto the preconditioned column, and the eluate was collected directly. The collected eluate was dried under a gentle stream of nitrogen. The dried residue was reconstituted in 1.0 mL of methanol–water solution (1:1, *v*/*v*) and filtered through a 0.22 μm nylon membrane. The filtrate was then subjected to UPLC-MS/MS analysis.

UPLC-MS/MS analyses were conducted on an LC-40AD system coupled with a triple quadrupole mass spectrometer (LCMS-8060NX, Shimadzu Corporation, Kyoto, Japan). Chromatographic separation was achieved on a Waters BEH C_18_ column (150 mm × 2.1 mm, 1.7 μm) with the column temperature maintained at 40 °C. The mobile phase consisted of 0.1% (*v*/*v*) formic acid in water (Phase A) and acetonitrile (Phase B), delivered at a constant flow rate of 0.3 mL/min. Gradient elution was employed with the following program: 0~1 min, 5% B; 1~8 min, linear gradient from 5% to 60% B; 8~10 min, linear gradient from 60% to 99% B; 10~13 min, isocratic elution at 99% B; 13~13.1 min, linear gradient from 99% back to 5% B. The injection volume was set at 2 μL.

The mass spectrometer was operated in electrospray ionization (ESI) with multiple reaction monitoring (MRM) for analyte detection. The optimized mass spectral parameters were as follows: ion source type, ESI^+^; nebulizing gas, nitrogen (3 L/min); drying gas, nitrogen (3 L/min); heating gas, air (15 L/min); collision gas, argon; interface voltage, 4.5 kV; interface temperature, 400 °C; de-solvation temperature, 650 °C; drift tube (DL) temperature, 250 °C; heating block temperature, 400 °C. Quantification of target analytes was performed using the internal standard method, and detailed mass spectral parameters for each analyte are provided in Supplementary Table S1.

### 2.3. Method Validation

The analytical method was validated to ensure its accuracy, precision, sensitivity, and reliability in accordance with established guidelines [14]. Linearity will be assessed by constructing multi-point calibration curves (e.g., 6~10 points) across the expected concentration range, with correlation coefficients (*R*^2^) typically required to exceed 0.990. Method detection limits (LODs) and limits of quantification (LOQs) will be determined based on signal-to-noise ratios (S/N of 3:1 for LOD and 10:1 for LOQ). Accuracy and precision will be evaluated through spiking experiments in blank matrices at different concentration levels (e.g., low and high), with spiking recoveries typically ranging from 70% to 120% and relative standard deviations (RSDs) below 10%. Matrix effects will be consistently monitored and compensated for using internal standards.

### 2.4. Risk Assessment

The estimated daily intake (EDI) will be calculated based on OPE concentrations in edible crustacean tissues, daily consumption rates, and average body weights for different age groups (e.g., children, teens, adults) as described in our previous reports [15,16,17]. Non-carcinogenic risks will be assessed using the target hazard quotient (THQ) and hazard index (HI) by comparing EDI with established reference doses (RfDs). Monte Carlo simulation (MCS) will be employed to conduct probabilistic risk assessments, accounting for uncertainties in exposure parameters and providing a range of potential risks.

The EDI, THQ, and HI were calculated as below (Equations (1)–(3))

(1) $$\mathrm{EDI}=\frac{C\times IR}{BW},$$

(2) $$\mathrm{THQ}=\frac{EDI}{RfD},$$

(3) $$\mathrm{HI}=\sum_{i=1}^{n}THQi,$$

C: mean concentration of individual OPE congener in crustaceans (μg/kg wet weight, μg/kg ww); IR: daily intake rate of crustaceans (g/day). Based on our previous report, the daily crustacean consumption rates were set as 11.85 g/day for children, 24.23 g/day for teens, and 33.32 for adults [18]; BW: body weight (kg). Reference values were 16.68 kg for children, 46.25 kg for teens, and 57.03 kg for adults, which are typical values for the general population in southeast China used in environmental health risk assessment studies [18].

EDI: Estimated daily intake of individual OPE congener (μg/kg body weight per day, μg/kg bw/day); RfD: Reference dose of individual OPE congener (μg/kg bw/day), representing the maximum daily intake of a pollutant that is unlikely to cause adverse health effects even with lifelong exposure. The data of RfDs were based on previous report [19]; THQi: THQ value of the OPE congener; n: Total number of detected OPE congeners. Risk classification criteria: THQ < 0.1 and HI < 1 indicate negligible non-carcinogenic risk; 0.1 ≤ THQ < 1 indicates low non-carcinogenic risk; THQ ≥ 1 or HI ≥ 1 indicates potential non-carcinogenic risk.

Monte Carlo simulation (MCS) was performed with 10,000 iterations using Oracle Crystal Ball software 10.0 (free-trial edition). The MCS results will output the mean of EDI, THQ, and HI, which can reflect the general scenario risks of the study population. This probabilistic assessment approach effectively reduces the uncertainty caused by fixed parameter values and improves the reliability of risk assessment results.

### 2.5. Statistical Analysis

Statistical analyses will be performed using OriginPro 2023 software (free-trial edition) and JMP 18.0 (free-trial edition). Descriptive statistics, including mean, median, range, standard deviation, and detection frequencies, will be calculated for all OPE concentrations. Data below the method detection limit (LOD) will be handled consistently, often by substituting with 0 for statistical calculations. Normality tests (e.g., Shapiro–Wilk test) were conducted, and non-parametric tests (Kruskal–Wallis test) were applied for comparisons between different groups or sampling sites. Spearman’s correlation analysis was employed to investigate relationships between OPE concentrations and environmental factors or other relevant parameters. Cluster analysis was used to identify inherent grouping patterns among OPE congeners based on their concentration characteristics. Statistical significance will be set at *p* < 0.05.

## 3. Results and Discussion

### 3.1. Validation of Instrumental Analytical Method

Method validation results demonstrated that the developed method exhibited good performance for the determination of 22 organophosphate esters (OPEs) (Supplementary Tables S2 and S3). All target analytes showed good linear relationships in their respective concentration ranges, with correlation coefficients (*R*^2^) ranging from 0.9979 to 0.9996. The limits of detection (LODs, S/N > 3) and limits of quantification (LOQs, S/N > 10) for the OPEs were in the ranges of 0.2~0.9 μg/kg and 0.6~2.7 μg/kg, respectively, with the lowest LOD (0.21 μg/kg) and LOQ (0.65 μg/kg) observed for TMPP. For accuracy and precision assessment, spiking experiments at low (5 μg/kg), medium (10 μg/kg), and high (50 μg/kg) concentrations yielded average recoveries of all OPEs between 78.3% (TnPP at low spiking) and 108.0% (TDBPP at medium spiking), with relative standard deviations (RSDs, *n* = 6) less than 10% across all spiking levels. These results collectively indicate that the method is linear, sensitive, accurate, and precise, making it suitable for reliable quantification of OPEs in the target matrix.

### 3.2. Distribution of OPEs in Crustaceans

#### 3.2.1. Total Concentrations and Species-Specific Differences

OPEs were widely detected in the three crustacean species from southeast China (Table 1). The total concentrations of 22 OPE congeners (ΣOPEs) in all samples ranged from 0 to 62.85 μg/kg ww, with a mean value of 5.74 μg/kg ww and a median of 1.49 μg/kg ww. This concentration level is comparable to OPE residues in crustaceans from other regions of China, such as crayfish from the Jianghan Plain (mean: 4.23 μg/kg ww) [10] and shrimp from the Beibu Gulf (mean: 7.12 μg/kg ww) [7], but lower than those from industrialized coastal areas (e.g., Pearl River Estuary, mean: 12.8 μg/kg ww) [4] (Sun et al., 2024), indicating moderate OPE pollution in southeast China’s crustacean habitats.

Notably, the mean of ΣOPEs in our study is lower than that in marine organisms from the Yellow River Estuary (median: 560 ng/g dw, equivalent to 56 μg/kg ww assuming 10% moisture content) [20], where intense petrochemical activities contribute to high OPE inputs. This discrepancy highlights the influence of regional industrial structures on OPE contamination levels. Compared with marine products from the Honghai Bay (mean: 55.8 ng/g dw, 5.58 μg/kg ww) [21], our results are consistent, confirming that crustaceans in South China generally carry moderate OPE residues.

Marine prawn had the highest mean ΣOPEs (6.52 μg/kg ww), followed by freshwater shrimp (5.80 μg/kg ww), while marine crab exhibited the lowest mean concentration (1.25 μg/kg ww). This variation may be attributed to differences in habitat, feeding behavior, and metabolic capacity among species. Marine prawn is typically a filtering feeder with a broader feeding range, leading to higher accumulation of waterborne and particle-associated OPEs [2]. Freshwater shrimp inhabit inland waters often influenced by anthropogenic activities (e.g., agricultural runoff, wastewater discharge), resulting in moderate OPE uptake. In contrast, marine crab are benthic omnivores with stronger metabolic detoxification capabilities for lipophilic contaminants [11], which may explain their lower OPE residues. This species-specific accumulation pattern aligns with findings from Laizhou Bay, where crustaceans showed higher OPE concentrations than some mollusk species due to distinct feeding strategies [22].

**Figure 2:**
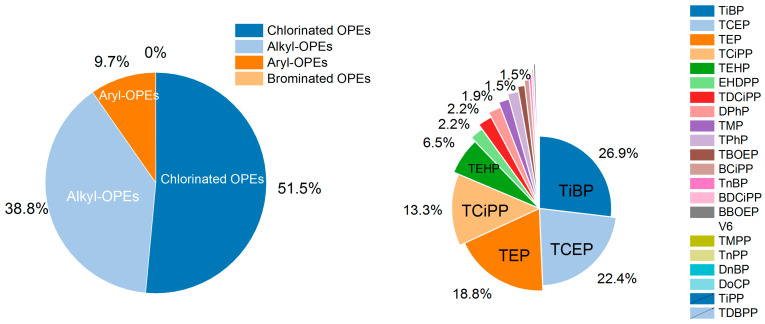
The percentage of different OPEs in crustaceans from southeast China.

As shown in Table 2 and Figure 2, among the 22 target OPE congeners in crustaceans from southeast China, chlorinated OPEs dominated (51.5% of ΣOPEs), led by tris(2-chloroethyl) phosphate (TCEP, mean 1.302 μg/kg ww, max 61.2 μg/kg ww) and tris(chloropropyl) phosphate (TCiPP, mean 0.773 μg/kg ww, max 15.8 μg/kg ww), followed by alkyl-OPEs dominated (38.8% of ΣOPEs) with tributyl phosphate (TiBP, mean 1.561 μg/kg ww, max 48.2 μg/kg ww) and triethyl phosphate (TEP, mean 1.089 μg/kg ww, max 23.6 μg/kg ww). Aryl-OPEs contributed minimally (9.7% of ΣOPEs) with diphenyl phosphate (DPhP), 2-ethylhexyl diphenyl phosphate (EHDPP), and triphenyl phosphate (TPhP) as the only detectable congeners (mean 0.087~0.129 μg/kg ww), while brominated OPEs (TDBPP) and some alkyl/chlorinated congeners (DoCP, TiPP) were not detected. TiBP, TEP, and TCEP accounted for 68.1% of ΣOPEs, with most congeners showing a median concentration of 0 μg/kg ww. TiBP and TEP’s dominance is linked to their extensive use as plasticizers in aquaculture equipment (e.g., nets, floats) and food contact materials, as well as industrial effluent discharge. This aligns with findings in coastal aquatic products from South China, where alkyl-OPEs are primary contaminants. TCEP and TCiPP’s prevalence, meanwhile, reflects their widespread application in flame retardants for building materials and textiles, with runoff transporting these persistent compounds to coastal waters [21].

#### 3.2.2. Congener Profiles

**Figure 3:**
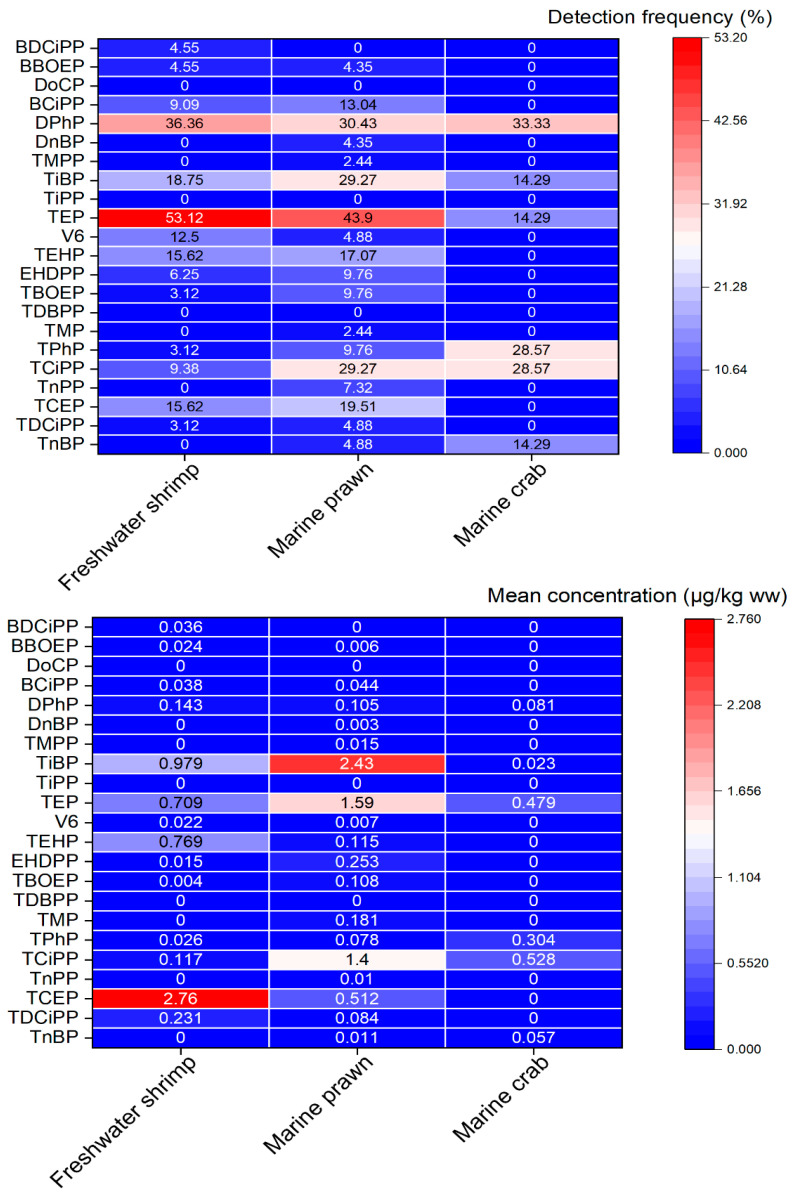
The mean concentration and detection frequency of different OPEs in crustaceans.

The compositional profiles of OPEs varied slightly among crustacean species but were dominated by three congeners: TiBP, TEP, and TCEP (Figure 3). TiBP was the most abundant OPE in marine prawn (mean: 2.43 μg/kg ww, 37.3% of ΣOPEs) and freshwater shrimp (mean: 0.979 μg/kg ww, 16.9% of ΣOPEs), which is consistent with its extensive use as a plasticizer in aquatic product packaging and aquaculture equipment [10]. TEP, a highly water-soluble OPE, was prevalent in all three species (mean: 1.089 μg/kg ww), accounting for 18.9% of ΣOPEs, reflecting its widespread release from industrial effluents and atmospheric deposition [5]. TCEP, a chlorinated OPE with high environmental persistence, was the dominant congener in freshwater shrimp (mean: 2.76 μg/kg ww, 47.6% of ΣOPEs), likely due to its extensive use in building materials and textile flame retardants, which are easily transported to freshwater ecosystems via runoff [6].

The dominance of chlorinated OPEs (TCEP, TCiPP) in freshwater shrimp is consistent with recent studies in urban aquatic environments. For example, TCEP and TCiPP accounted for 56% and 34% of ΣOPEs in indoor air of Guangzhou homes and offices [23], and their subsequent runoff may contaminate adjacent freshwater systems. In contrast, aryl-OPEs such as triphenyl phosphate (TPhP) and its metabolite diphenyl phosphate (DPhP) were detected at low concentrations in our samples, which differs from the Honghai Bay study where TPhP was one of the most frequently detected congeners [21]. This difference may be caused by regional variations in OPE usage. Southeast China’s manufacturing sector relies more on alkyl and chlorinated OPEs, while aryl-OPEs are more commonly used in coastal textile industries.

The detection frequencies of OPE congeners varied significantly among species (Figure 3). TEP had the highest overall detection rate (53.12% in freshwater shrimp, 43.9% in marine prawn, 14.29% in marine crab), followed by DPhP (36.36~33.33%) and TiBP (18.75~29.27%). Chlorinated OPEs (e.g., TCEP, TCiPP) had moderate detection rates (9.38~29.27%), consistent with their persistence in aquatic environments [8]. Notably, several congeners (e.g., DoCP, TiPP, TDBPP) were not detected in any samples, possibly due to their limited use in southeast China or low environmental mobility. The higher detection frequency of OPEs in freshwater shrimp and marine prawn suggests that these species are more sensitive to OPE contamination and suitable as bioindicators for environmental monitoring.

The high detection rate of DPhP (33.33~36.36%) across all species merits attention, as it is a major metabolite of aryl-OPEs and exhibits higher toxicity than its parent compounds in some aquatic organisms [24]. Additionally, the detection of TBOEP in 12.5% of marine prawn samples aligns with its high usage in plasticizers and hydraulic fluids, and recent human biomonitoring studies have identified TBOEP as a major contributor to non-carcinogenic risk [25], emphasizing the need for continued surveillance.

### 3.3. Spatial and Temporal Variations in OPEs

#### 3.3.1. Spatial Distribution

**Figure 4:**
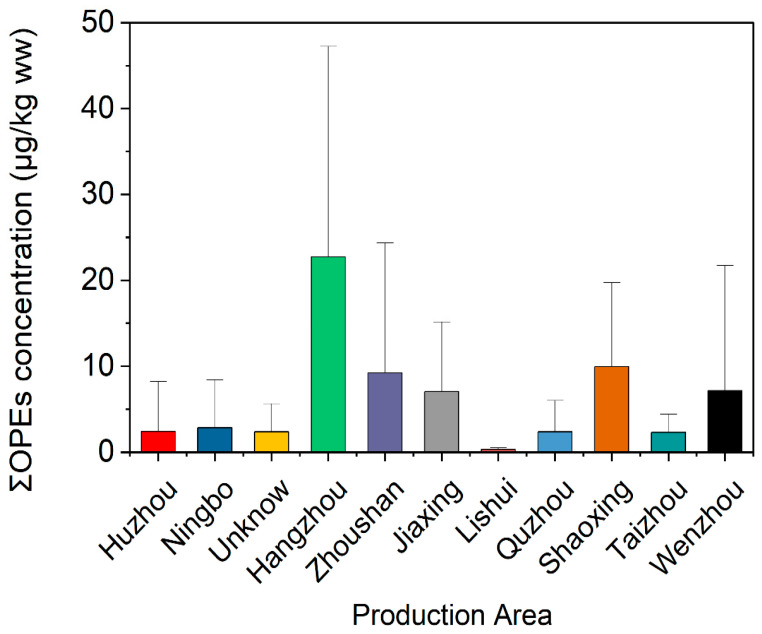
Concentrations of ΣOPEs in crustaceans from different production areas in southeast China.

The spatial distribution of ΣOPEs in crustaceans from 11 production areas in southeast China showed significant regional differences (Figure 4). Zhoushan (mean: 15.3 μg/kg ww) and Taizhou (mean: 12.7 μg/kg ww) had the highest ΣOPE concentrations, followed by Ningbo (mean: 8.9 μg/kg ww) and Wenzhou (mean: 7.8 μg/kg ww). These areas are major coastal cities with intensive industrial activities (e.g., electronics manufacturing, shipbuilding) and aquaculture operations, leading to high OPE inputs from wastewater discharge and plastic waste leaching [7]. In contrast, Quzhou (mean: 1.2 μg/kg ww) and Lishui (mean: 1.5 μg/kg ww), located in inland mountainous regions, had the lowest OPE levels, reflecting minimal anthropogenic disturbance.

The high OPE levels in Zhoushan and Taizhou are consistent with their status as key marine aquaculture hubs. A recent study on plastic greenhouses showed that agricultural plastic films are important sources of OPEs, with migration to surrounding soils and water bodies [26]. Similarly, the extensive use of plastic nets, floats, and packaging in coastal aquaculture of southeast China may contribute significantly to local OPE contamination. Additionally, these cities are part of the Yangtze River Delta economic zone, where industrial wastewater discharge is a major source of OPEs [27].

The spatial variation in OPE congener profiles also indicated regional pollution sources. For example, TCEP was predominant in Hangzhou and Jiaxing (accounting for 40~50% of ΣOPEs), which are influenced by textile and chemical industries. TiBP was the dominant congener in Zhoushan and Taizhou (35~40% of ΣOPEs), associated with extensive aquaculture and seafood processing activities. These results suggest that local industrial structure and human activities are key factors driving the spatial distribution of OPEs in crustaceans. Comparable spatial patterns were observed in the Yellow River Estuary, where petrochemical industrial clusters correlated with high OPE concentrations in marine organisms [20].

#### 3.3.2. Temporal Variations

**Figure 5:**
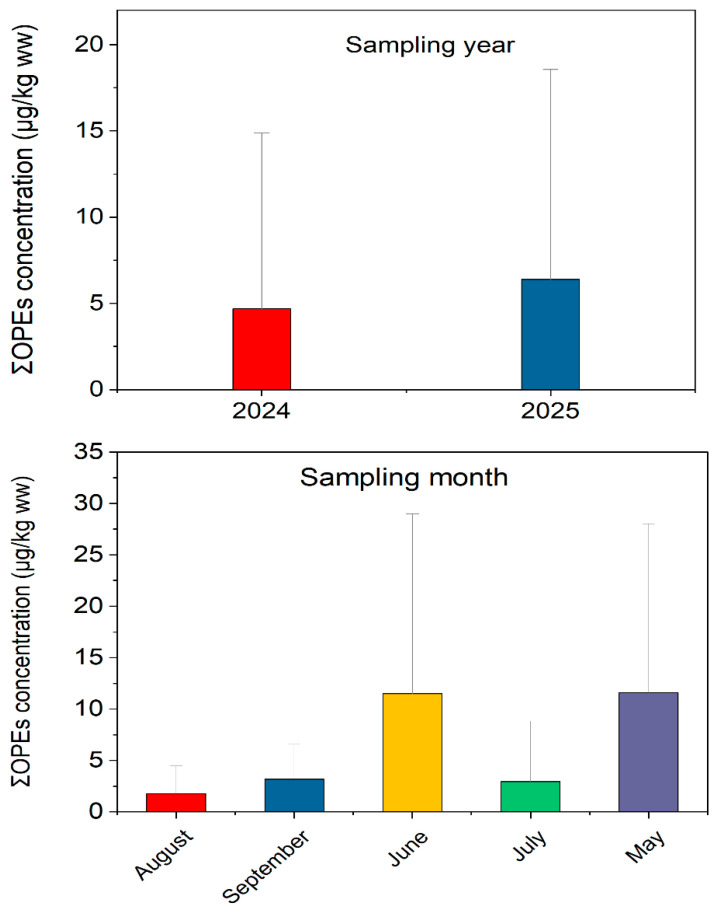
The change in ΣOPEs concentrations in different sampling months and years.

Temporal variations in ΣOPEs in crustaceans were analyzed across sampling months (May–September 2024) and years (2024~2025) (Figure 5). No significant seasonal differences were observed (Kruskal–Wallis test, *p* > 0.05), with mean ΣOPE concentrations ranging from 4.8 μg/kg ww (May) to 6.5 μg/kg ww (August). This is consistent with previous studies in the Yangtze River basin, where OPE concentrations showed no obvious seasonal fluctuations due to continuous anthropogenic inputs [5]. However, a slight increase in ΣOPEs was observed in August, possibly due to enhanced volatilization and leaching of OPEs from plastic materials under high temperatures [24]. No significant interannual differences were found between 2024 and 2025 (*p* > 0.05), indicating stable OPE pollution levels in southeast China’s crustacean habitats over the study period.

The lack of significant seasonal variation contrasts with some estuarine studies, where rainfall-driven runoff increases OPE inputs in wet seasons. For example, in the Liao River estuary, seasonal differences in OPE concentrations were attributed to river discharge [28]. The stable seasonal levels in our study may reflect the combined effects of continuous industrial emissions and aquaculture activities, which offset the impact of seasonal runoff. Additionally, the thermal stability of most OPEs may minimize temperature-driven degradation, contributing to consistent residue levels across seasons [12].

### 3.4. Correlation and Hierarchical Cluster Analysis (HCA)

#### 3.4.1. Correlation Analysis

**Figure 6:**
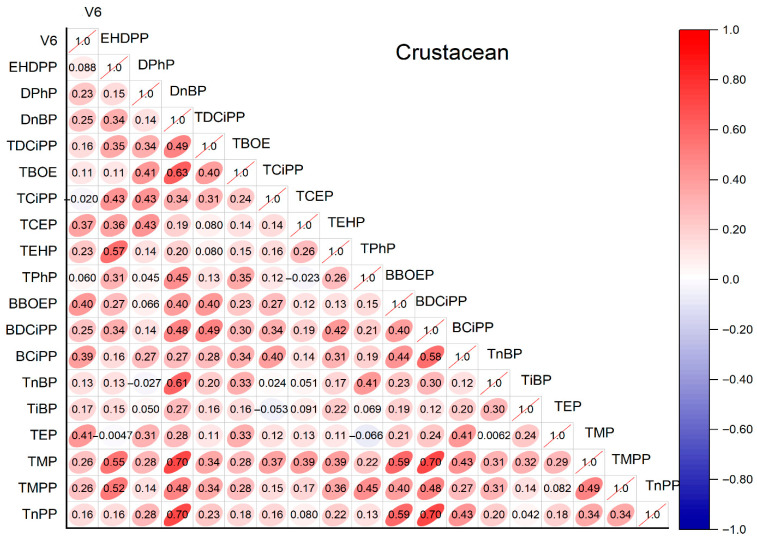
The correlation of these OPEs in crustaceans from southeast China.

Spearman’s correlation analysis was conducted to explore the relationships between OPE congeners (Figure 6). Strong positive correlations were observed between TMPP and TMP (*r* = 0.70, *p* < 0.01), TDBPP and DoCP (*r* = 0.63, *p* < 0.01), and TCiPP and TCEP (*r* = 0.43, *p* < 0.01). These correlations suggest common pollution sources for these congeners, such as industrial emissions and wastewater discharge [6]. For example, TMPP and TMP are both used as plasticizers in electronic products, while TCiPP and TCEP are typical chlorinated OPEs from flame retardants. In contrast, weak or negative correlations were found between highly water-soluble OPEs (e.g., TEP) and lipophilic OPEs (e.g., TEHP) (*r* = −0.02, *p* > 0.05), reflecting differences in their environmental transport and accumulation pathways. The positive correlation between TCiPP and TCEP is consistent with their co-occurrence in industrial products and has been reported in multiple aquatic environments, including the East China Sea and Yellow Sea [29] and indoor air [23]. Additionally, the moderate correlation between TiBP and TnBP (*r* = 0.38, *p* < 0.05) supports their common use as plasticizers in aquaculture equipment and packaging materials [10]. These correlations align with the trophic transfer study in Laizhou Bay, where structurally similar OPEs showed consistent accumulation patterns in marine food webs [25].

#### 3.4.2. Hierarchical Cluster Analysis (HCA)

**Figure 7:**
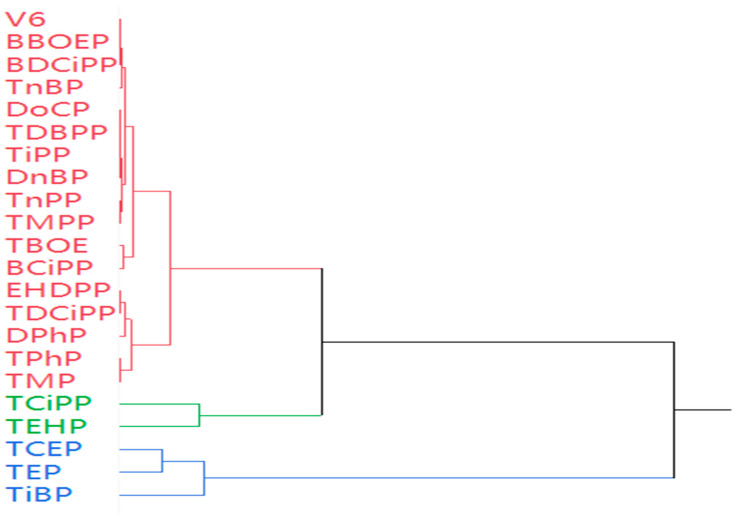
The hierarchical cluster of these OPEs in crustaceans from southeast China.

HCA was performed to identify pollution source clusters (Figure 7). The 22 OPE congeners were divided into three main clusters: Cluster 1 included TEP, TCEP, and TCiPP (chlorinated and water-soluble OPEs), indicating industrial and wastewater sources; Cluster 2 contained TiBP, TnBP, and TBOEP (alkyl-OPEs), associated with plasticizers and aquaculture activities; Cluster 3 comprised TPhP, DPhP, and EHDPP (aryl-OPEs), reflecting degradation products and textile industry inputs. This clustering is consistent with the pollution source characteristics of southeast China, where industrial emissions, plastic waste, and aquaculture are the main contributors to OPE contamination [13].

Cluster 1 (industrial-derived OPEs) aligns with the principal component analysis results from the Yellow River Estuary, where petrochemical pollution and industrial wastewater were identified as major OPE sources [20]. Cluster 2 (aquaculture-related OPEs) is supported by the recent finding that plastic greenhouse materials are key sources of alkyl-OPEs in agricultural and aquatic environments [26] (Zhang et al., 2024).

### 3.5. Risk Assessment

Human health risks from dietary exposure to OPEs through crustacean consumption were assessed using the estimated daily intake (EDI), target hazard quotient (THQ), and hazard index (HI) (Table 3). The EDI values of OPEs for adults, teens and children ranged from 0.003 to 0.675 μg/kg bw/day and were well below the reference doses (RfD) recommended by the US EPA (e.g., TCEP: 22 μg/kg bw/day; TEP: 125 μg/kg bw/day). The THQ values of individual OPEs were all less than 0.1 for all groups, and the HI values (sum of THQs) with 0.046~0.6 are all below 1, indicating negligible non-carcinogenic risks. However, frequent seafood consumers may have slightly higher exposure, which needs long-term monitoring to avoid potential cumulative risks.

Our risk assessment is consistent with recent studies on Chinese populations. For example, a large-scale biomonitoring study of 1869 urban residents found that OPE exposure was widespread, but the overall HI was <1, with TBOEP being the major risk contributor [25]. Similarly, in the Honghai Bay, OPEs in marine products posed acceptable non-carcinogenic and carcinogenic risks to residents of all age groups [21]. However, emerging evidence indicates that certain OPEs may have previously unrecognized health effects. For example, aryl-OPEs, bisphenol a bis (diphenyl phosphate) (BDP) and cresyl diphenyl phosphate (CDP), can disrupt hemostatic balance via PPARγ-mediated pathways [30], and gestational exposure to chlorinated OPEs may affect neonatal development due to their high transplacental transfer efficiency [31]. These findings highlight the need to expand risk assessment frameworks to include emerging toxic endpoints beyond traditional non-carcinogenic and carcinogenic effects.

It should be noted that dietary exposure via crustacean consumption is only one component of total OPE exposure. Indoor air inhalation is another major pathway, with private cars and offices showing high OPE concentrations [32]. For coastal populations in southeast China, the combined exposure from diet, inhalation, and dermal contact may increase overall health risks, particularly for vulnerable groups such as pregnant women and children. Future risk assessments should adopt a multi-pathway approach to provide a more comprehensive evaluation.

## 4. Conclusions

This study comprehensively investigated the residue levels, distribution characteristics, and exposure risks of OPEs in crustaceans from southeast China. The key findings are as follows: (1) OPEs were widely detected in freshwater shrimp, marine prawn, and marine crab, with mean ΣOPE concentrations of 5.80, 6.52, and 1.25 μg/kg ww, respectively; (2) TiBP, TEP, and TCEP were the dominant congeners, which indicates the influence of industrial emissions, plastic waste, and aquaculture activities; (3) Spatial variations showed higher OPE levels in coastal industrial cities (Zhoushan, Taizhou) than in inland regions, while no significant temporal differences were observed; (4) Human health risk assessment revealed negligible non-carcinogenic and carcinogenic risks from crustacean consumption.

It provides valuable data for understanding OPE pollution in southeast China’s aquatic ecosystems and supports food safety regulation. However, limitations exist, such as the small number of sampling sites in some regions and the lack of data on OPE metabolites. Future research should expand the sampling range, include more crustacean species, and investigate the synergistic effects of OPEs with other pollutants (e.g., nanoparticles, microplastics), and water management should consider the possible pollution [33,34]. Given the increasing use of emerging OPEs and their potential toxic effects, long-term monitoring programs and updated risk assessment frameworks are urgently needed.

## Figures and Tables

**Table 1:** The distribution of ΣOPEs in crustaceans from southeast China.

Type	N Total	Concentration (μg/kg ww)			
		Mean	Minimum	Median	Maximum
Total		5.74	0	1.49	62.85
Freshwater shrimp	239	5.80	0	1.35	62.85
Marine crab	84	1.25	0	0.00	6.41
Marine prawn	210	6.52	0	2.20	56.13

**Table 2:** The concentration of different OPEs in crustaceans from southeast China.

Type	Compound	Concentration (μg/kg ww)			
		Mean	Minimum	Median	Maximum
chlorinated OPEs	BDCiPP	0.015	0	0	0.804
alkyl-OPEs	BBOEP	0.013	0	0	0.524
alkyl-OPEs	DoCP	0.000	0	0	0
chlorinated OPEs	BCiPP	0.036	0	0	0.477
aryl-OPEs	DPhP	0.111	0	0	1.48
alkyl-OPEs	DnBP	0.001	0	0	0.067
aryl-OPEs	TMPP	0.007	0	0	0.627
alkyl-OPEs	TiBP	1.561	0	0	48.2
alkyl-OPEs	TiPP	0.000	0	0	0
alkyl-OPEs	TEP	1.089	0	0	23.6
chlorinated OPEs	V6	0.012	0	0	0.476
alkyl-OPEs	TEHP	0.375	0	0	23.0
aryl-OPEs	EHDPP	0.129	0	0	4.29
alkyl-OPEs	TBOEP	0.054	0	0	2.84
brominated OPEs	TDBPP	0.000	0	0	0
alkyl-OPEs	TMP	0.088	0	0	7.42
aryl-OPEs	TPhP	0.087	0	0	1.39
chlorinated OPEs	TCiPP	0.773	0	0	15.8
alkyl-OPEs	TnPP	0.005	0	0	0.170
chlorinated OPEs	TCEP	1.302	0	0	61.2
chlorinated OPEs	TDCiPP	0.129	0	0	7.39
alkyl-OPEs	TnBP	0.020	0	0	0.814

**Table 3:** The results of EDIs and THQs for different OPE residues in crustaceans from southeast China.

Compound	Mean (μg/kg ww)	RfD	Children		Teens		Adults	
			EDI	THQ	EDI	THQ	EDI	THQ
TMPP	0.007	13	0.004	0.00026	0.003	0.00021	0.003	0.00023
TEP	1.089	125	0.581	0.00465	0.449	0.00359	0.495	0.00396
TEHP	0.375	35	0.256	0.00732	0.188	0.00537	0.211	0.00602
TBOEP	0.054	15	0.03	0.00203	0.023	0.00154	0.025	0.00168
TMP	0.088	10	0.05	0.00508	0.039	0.00386	0.043	0.00435
TPhP	0.087	70	0.046	0.00066	0.035	0.0005	0.040	0.00058
TCiPP	0.773	80	0.388	0.00485	0.298	0.00372	0.336	0.0042
TCEP	1.302	22	0.675	0.03067	0.523	0.02378	0.572	0.026
TDCiPP	0.129	15	0.065	0.00432	0.051	0.00338	0.057	0.00378
TnBP	0.02	24	0.012	0.0005	0.009	0.00037	0.01	0.00042

Note: The unit of RfD is μg/kg bw/day, and unit of EDI is μg/kg bw/day. The values of EDI and THQ were the median data from Monte Carlo simulation.

## Data Availability

The original contributions presented in this study are included in the article/Supplementary Material. Further inquiries can be directed to the corresponding authors.

## Supplementary Materials

The following supporting information can be downloaded at: https://www.mdpi.com/article/10.3390/jox16020058/s1, Table S1: The mass parameters of 22 OPEs; Table S2: The linear equation, LOD, and LOQ for OPEs; Table S3: The spiking recoveries and RSDs for OPEs.
