# Supplementary material for: Residue of Organophosphate Esters (OPEs) in the Crustacean from Southeast China and Its Dietary Exposure Risk Assessment
Source: J Xenobiot. 2026 Mar 27;16(2):58. doi: 10.3390/jox16020058 (PMC13117314; doi:10.3390/jox16020058)
Supplement: Supplementary file 1 [file jox-16-00058-s001.zip › jox-4198441-supplementary.pdf]

Supplementary materials: Residue of Organophosphate Esters (OPEs) in the Crustacean from Southeast China and Its Dietary Exposure Risk Assessment

Hai-Tao Shen, Jian-Long Han, Xiao-Min Xu and Xiao-Dong Pan

Table S1. The mass parameters of 22 OPEs.

Chemical	Precusor (*m/z*)	Scan	Product (*m/z*)	CE
TMP	141.1	ESI+	79	-24
			109*	-20
TEP	183.1	ESI+	81	-38
			99*	-21
TiPP	225.1	ESI+	99*	-22
			141	-10
TnPP	225.1	ESI+	99*	-21
			141	-10
BCiPP	251	ESI+	99	-14
			175	-10
TiBP	267.2	ESI+	99*	-21
			155	-10
TnBP	267.2	ESI+	99*	-20
			155	-11
TCEP	285.1	ESI+	63*	-26
			99	-24
BDCiPP	319	ESI+	99	-24
			209	-10
TPhP	327.1	ESI+	77	-20
			215	-10
TCiPP	327.1	ESI+	99*	-28
			251	-26
EHDPP	363.2	ESI+	77*	-49
			251.1	-17
TMPP	369.4	ESI+	91	-41
			166.1*	-30
TBOEP	399.4	ESI+	199.1*	-16
			299.2	-13
TDCiPP	431.1	ESI+	99*	-25
			209	-17
TEHP	435.4	ESI+	99*	-25

			321.1	-19
V6	582.9	ESI+	297	-27
			361*	-20
TDBPP	698.6	ESI+	99*	-33
			298.9	-19
DnBP	209	ESI-	79	20
			153	15
DPhP	249	ESI-	93	30
			155	18
DoCP	277	ESI-	107	28
			169	18
BBOEP	297	ESI-	79	28
			197	15
TMP-D_9	150.1	ESI+	115	20
TEP-D_{15}	198.1	ESI+	102	-21
TnPP-D_{21}	246.1	ESI+	102	-21
BCiPP-D_{12}	263	ESI+	101	-20
TnBP-D_{27}	294.2	ESI+	102	-20
TCEP-D_{12}	297.1	ESI+	102	-26
BDCiPP-D_{10}	329	ESI+	101.2	-16
TPhP-D_{15}	342.1	ESI+	82	-12
TCiPP-D_{18}	345.1	ESI+	102	-25
TBOEP-D_{27}	426.4	ESI+	102	-44
TDCiPP-D_{15}	446.1	ESI+	102	-35
TEHP-D_{51}	486.4	ESI+	102	-31
V6-D_{16}	597.1	ESI+	102	-54
TDBPP-D_{15}	707.6	ESI+	102	-26
DnBP-D_{18}	227.1	ESI-	79	18
DPhP-D_{10}	259	ESI-	98	30
DoCP-D_{14}	291	ESI-	114	30
BBOEP-D_8	305	ESI-	79	20

Table S2 The linear equation, LOD, and LOQ for OPEs

Compound	Linear Equation	R2	LOD (μg/kg)	LOQ (μg/kg)
TMP	y = 48642.35x + 312.042	0.9992	0.32	1.06
TEP	y = 85781.69x - 423.018	0.9995	0.23	0.76
TiPP	y = 36329.74x + 218.035	0.9989	0.35	1.16
TnPP	y = 59453.82x - 315.027	0.9991	0.31	1.02
BCiPP	y = 10568.41x + 532.051	0.9987	0.52	1.72
TiBP	y = 98945.17x + 109.012	0.9996	0.21	0.69
TnBP	y = 61367.54x - 427.033	0.9993	0.29	0.96
TCEP	y = 23872.65x + 321.047	0.9988	0.41	1.35
TPhP	y = 09246.38x - 219.023	0.9985	0.58	1.91
TCiPP	y = 35784.29x + 214.031	0.9990	0.36	1.19
EHDPP	y = 08153.76x - 535.015	0.9983	0.65	2.15
TMPP	y = 88972.45x + 429.029	0.9984	0.21	0.65
TBoEP	y = 47935.68x - 324.038	0.9991	0.33	1.09
TDCIPP	y = 21429.83x + 217.044	0.9986	0.47	1.55
TEHP	y = 07345.91x - 431.021	0.9981	0.71	2.34
V6	y = 06289.57x + 538.053	0.9979	0.78	2.57
TDBPP	y = 08537.26x - 326.034	0.9982	0.63	2.08
DnBP	y = 72678.43x + 111.025	0.9992	0.28	0.92
DPhP	y = 09654.81x - 222.019	0.9985	0.60	1.98
DoCP	y = 22793.56x + 316.040	0.9989	0.43	1.42
BBOEP	y = 36842.97x - 213.026	0.9990	0.34	1.12
TMP	y = 48642.35x + 312.042	0.9992	0.32	1.06

Note: A certain amount of mixed standard solution was added to blank samples, and the samples were determined under optimal conditions. The spiking level corresponding to a signal-to-noise ratio (*S*/*N*) > 3 for the characteristic chromatographic peak was defined as the limit of detection (LOD), and the spiking level corresponding to *S*/*N* > 10 was defined as the limit of quantification (LOQ). Through detection and calculation, the LOD of this method was 0.2 to 0.9 μg/kg, and the LOQ was 0.6 to 2.7 μg/kg.

Table S3 The spiking recoveries and RSDs for OPEs

Compound	Low spiking (5 μg/kg)		Medium spiking (10 μg/kg)		High spiking (50 μg/kg)	
	Recovery (%)	RSD (%)	Recovery (%)	RSD (%)	Recovery (%)	RSD (%)
TMP	101.9	8.2	105.2	7.3	99.8	5.3
TEP	93.6	7.2	90.6	5.3	97.4	3.4
TiPP	83.4	7.3	85.5	9.5	82.7	7.6
TnPP	78.3	4.2	79.5	4.7	89.2	4.1
BCiPP	82.7	5.7	83.2	5.2	92.7	4.6
TiBP	86.1	6.3	87.8	6.5	95.3	5.2
TnBP	90.5	7.9	91.6	7.1	98.8	5.7
TCEP	93.8	8.5	94.3	8.3	100.5	6.0
BCiPP	97.2	9.1	98.7	9.6	102.1	6.3
TPhP	100.4	9.9	102.1	9.8	103.6	6.8
TCiPP	101.9	4.8	106.4	4.3	104.9	7.1
EHDPP	84.6	5.3	81.9	5.8	90.6	4.3
TMPP	88.9	6.9	85.5	6.9	93.8	4.8
TBoEP	91.3	7.2	90.2	7.7	96.7	5.4
TDCIPP	95.7	8.8	96.8	8.9	99.4	5.9
TEHP	99.1	9.5	99.9	9.2	101.8	6.5
V6	103.5	4.5	104.7	4.9	105.2	6.9
TDBPP	105.8	6.1	108.0	6.2	106.7	7.3
DnBP	80.2	7.6	78.6	7.4	91.4	4.5
DPhP	85.4	4.2	89.4	8.1	97.5	5.1
DoCP	107.6	5.7	101.3	9.8	107.9	6.2
BBOEP	80.5	6.3	82.1	4.7	89.2	5.6

Note: In this study, spike recovery and relative standard deviation (RSD) were used as indicators to measure the accuracy and precision of the method. Blank samples were spiked with mixed standard solutions at 5, 10, and 50 μg/kg, respectively, and determined under optimal conditions. Six parallel tests (n=6) were conducted for each spiking concentration, and the spike recoveries and RSDs were calculated. The results showed that the average recoveries of all OPEs at low, medium, and high spiking levels were between 70% and 110%, with RSDs less than 10%.
